# Prognostic significance of lactate/albumin ratio in respiratory failure and sepsis

**DOI:** 10.1080/07853890.2025.2482024

**Published:** 2025-03-25

**Authors:** Chandra Prasad Acharya, Alok Yadav, Saugat Pokhrel, Sanjib Bastola, Suprabha Jha

**Affiliations:** Internal Medicine, Manipal College of Medical Sciences, Pokhara, Nepal

**Keywords:** Lactate, albumin, sepsis, ARF, LAR, predictor, mortality, hospital-outcome

## Abstract

**Introduction:**

Prognostic markers like SOFA and APACHE-II scores for sepsis and acute respiratory failure (ARF) are often complex for routine use. This study evaluated the lactate-albumin ratio (LAR) as a prognostic marker for in-hospital outcomes, mechanical ventilation, and inotrope requirement.

**Methods:**

A prospective cohort study was conducted among ICU and Internal Medicine Unit patients at Manipal Teaching Hospital, approved by the Institutional Review Committee (approval number IRC/MCOMS/584). Arterial samples for ABG values (lactate, PaO2, PaCO2, HCO3, and FiO2) and venous samples for albumin, bilirubin and creatinine were collected on admission. Arterial lactate, serum albumin, LAR and SOFA scores were recorded and compared with in-hospital outcomes. Statistical analyses were performed using SPSS version 25, with ROC-AUC for assessing prognostic markers (LAR, lactate alone) and Delong’s test for comparison.

**Result:**

Among 115 sepsis cases, ROC-AUC of LAR for in-hospital mortality (cut-off 1.78), mechanical ventilation (1.28), and inotropes (1.07) were 0.914, 0.881, and 0.819, respectively. Among 99 ARF cases, ROC-AUC for mortality (1.98), ventilation (1.10), and inotropes (1.18) were 0.878, 0.958, and 0.876. Among 43 sepsis + ARF cases, ROC-AUC for mortality (2.14), ventilation (1.20), and inotropes (1.20) were 0.853, 0.874, and 0.849.

**Conclusion:**

The lactate-albumin ratio was a better prognostic marker than albumin alone and SOFA score for predicting in-hospital mortality, need for mechanical ventilation and inotropes in sepsis, ARF and combined Sepsis and ARF patients whereas it was statistically equivalent to lactate alone in predicting in hospital outcome. Lactate-albumin ratio also indicated disease progression, where an increase in cut-off value was seen with progressed or severe disease.

## Introduction

Sepsis is defined as a life-threatening organ dysfunction caused by a dysregulated host response to infection. The diagnosis of sepsis is based on organ dysfunction which can be identified as an acute change in the total Sequential Organ Failure Assessment (SOFA) score of ≥ 2 points consequent to the infection [1]. Although there are prognostic markers such as SOFA and Acute Physiology and Chronic Health Evaluation (APACHE – II) scores for sepsis, they are often too sophisticated for daily practice. Thus, a simpler yet effective predictive marker for in-hospital outcomeslikethe requirement of inotropes, need for mechanical ventilation, and mortality among sepsis patients, is required.

The lactate/albumin ratio(LAR), an easy-to-calculate marker, has been found to correlate with the development of multiple organ dysfunction syndrome, as well as predict ICU and in-hospital mortality and long-term mortality in sepsis and septic shock [2–5]. The lactate/albumin ratio was comparatively high in patients requiring mechanical ventilation and may be beneficial for identifying high-risk patients [3,5,6]. The lactate/albumin ratio was even found to be prognostically superior to lactate alone, albumin alone, and lactate clearance in predicting in-hospital mortality, requirement for renal replacement therapy, length of ICU stay, and duration of mechanical ventilation [7–10]. However, the effectiveness of LAR as a predictive marker in comparison with the SOFA score is not well established.

Lactate is a byproduct of anaerobic metabolism, often used as biomarker in sepsis and critical care. Elevated lactate levels indicate tissue hypoperfusion and hypoxia, which are common in conditions like sepsis and acute respiratory failure (ARF) [11–13]. Albumin is a negative acute phase reactant (APR) and protein produced by liver. Albumin decreases in serum levels during inflammation, especially in conditions like acute respiratory failure and sepsis. This reduction is due to decreased hepatic production and increased proteolysis, as the body redirects amino acids to produce positive APRs that help combat inflammation [14–16]. This is the rationale behind raised LAR during sepsis and ARF.

Acute respiratory failure(ARF) is defined by an arterial oxygen tension(PaO_2_) of <8.0 kPa (60 mmHg), arterial carbon dioxide tension (PaCO_2_) of >6.0 kPa (45 mmHg), or both [17]. The significance of lactate and albumin in acute respiratory failure (ARF) has not been studied. However, in the context of respiratory diseases such as Acute Respiratory Distress Syndrome (ARDS) and COVID-19, lactate was found to be a good prognostic marker for guiding resuscitation, and predicting short-term mortality [18–20]. Low serum albumin levels have been associated with a high risk of ARF requiring mechanical ventilation and prolonged ICU stay [21–23]. A high serum albumin level is also associated with fewer adverse outcomes, less ARDS development, and less ICU admission in COVID-19. The prognostic lactate/albumin ratio (LAR) was found to be superior to lactate alone, albumin alone, and SOFA in predicting 30-day mortality [24]. Even a reduction of one unit of LAR was associated with a significant reduction in mortality [25].

This study focused on the effectiveness of LAR in predicting in-hospital mortality, the requirement of inotropes, and the need for mechanical ventilation among ARF cases regardless of the cause. However, no studies have explored the role of LAR as a predictive marker for in-hospital outcomes in patients diagnosed with both sepsis and ARF.

This study aims to analyze the prognostic significance of Lactate/Albumin ratio for in-hospital complications and outcomes among patients diagnosed with Acute Respiratory failure, the prognostic significance of LAR for in-hospital complications and outcomes among patients diagnosed with sepsis. This is a novel study which aims at demonstrating the effectiveness of LAR as prognostic marker in patients who are suffering from both sepsis and ARF as per above mentioned definition of sepsis and ARF respectively [1,11]. By assessing the combined effect of these two critical conditions, this research aims to provide valuable insights into how LAR can predict patient outcomes including the need for mechanical ventilation, the need for inotropes and mortality. Thus, identifying a reliable prognostic tool can guide clinical decision making for early necessary interventions

## Methodology

A prospective cohort observational study was conducted among patients admitted to the ICU and Internal medicine unit of the Manipal Teaching Hospital diagnosed with ARF, Sepsis, or both after taking informed written consent. The study protocol, including the informed written consent form, was reviewed and approved by the Institutional Review Committee(IRC) of Manipal College of Medical Sciences. The IRC provided approval under the ethical board approval number IRC/MCOMS/584. The patients were observed on the day of admission, and vital signs at presentation were noted. Arterial blood samples were collected at the time of admission for arterial blood analysis (ABG) to obtain values for arterial lactate, PaO_2_, PaCO_2_, HCO_3_, and FiO_2_. Venous blood samples were collected to determine serum albumin, bilirubin, and creatinine. Arterial lactate level, serum albumin level, LAR, and SOFA score were recorded at the time of admission. Patients were regularly monitored until discharge, and in-hospital outcomes (alive/death), the need for inotropes, mechanical ventilation, and length of hospital stay were recorded and divided into three groups:

- Patients diagnosed with sepsis as per SOFA criteria.
- Patients diagnosed with ARF.
- Patients diagnosed with both sepsis and ARF.

The role of LAR as a predictive marker for in-hospital mortality, length of hospital stay, need of mechanical ventilation, and the requirement for inotropes was analyzed using various statistical method as per SPSS version 25.

Sample size:

For sepsis patient:

Incidence of Sepsis (*p*): 13.6–39.3% (27%) [26]
*q* (100 − *p*) = 73%
*Z* = status of level of confidence = 1.96
*e* = absolute allowable error = 10%
*n* = *z*^2^ × *p* × *q*/*e*^2^
= 1.96^2^ × 27 × 73/100
= 75.71 = approx. 76 cases
For Acute Respiratory failure
*P* = incidence of acute respiratory failure = 32% [17]
*q* = 100 – *p* = 100 – 32 = 68
*z* = level of confidence interval = 1.96
*z* = maximum allowable error = 10%
*n* = *z*^2^ × *p* × *q*/*e*^2^
= 1.96^2^ × 32 × 68/10^2^
= 83.59 = approx. 84 cases.

Inclusion criteria

1. Patients diagnosed with sepsis as per SOFA score.
2. Patients diagnosed with ARF.

Exclusion criteria

1. Patients unwilling to participate or provide consent.
2. Patients with preexisting liver disease or chronic liver disease.

### Statistical analysis

Statistical analyses were performed using SPSS version 25. To analyze the predictive nature and to find cut off values for LAR, lactate alone, albumin alone and SOFA Score for the need of inotropes, mechanical ventilation and mortality ROC-AUC curve and youden index analysis were conducted respectively. Other tests included an independent sample t- test, correlation and regression analysis as required.Delong test was used to compare ROC-AUC curve and *p* value < 0.05 was consider statistically significant.

## Results

A total of 115 patients with sepsis, 99 with ARF, and 43 with both sepsis and ARF were included in the study. Among the patients with sepsis, 59 were male and 56 were female. In the ARF group, 44 were male and 55 were female. Of those with both sepsis and ARF, 19 were male and 24 were female. The mean age of patients with sepsis was 63.43 ± 18.67 years, for ARF it was 65.42 ± 18.65 years, and for those with both conditions, it was 61.23 ± 19.61 years. In the sepsis group, 75 patients survived, 27 died, and 13 left against medical advice (LAMA). In the ARF group, 67 survived, 25 died, and 7 left against medical advice. In the group with both sepsis and ARF, 20 survived, 17 died, and 6 left against medical advice. Inotropes were used in 70 patients with sepsis, 52 patients with ARF, and 31 patients with both conditions. In the sepsis group, 45 patients did not require inotropes, 46 in the ARF group, and 12 in the group with both conditions. Mechanical ventilation was required in 54 patients with sepsis, 43 patients with ARF, and 31 patients with both sepsis and ARF. Mechanical ventilation was not required in 61 patients with sepsis, 56 patients with ARF, and 12 patients with both conditions. Patients with sepsis had a mean duration of mechanical ventilation of 1.83 days, 1.58 days for ARF patients, and 2.84 days for those with both conditions. Surviving sepsis patients had a mean hospital stay of 8.48 days, while those who died had a mean stay of 6.37 days (*p* = 0.015). For ARF patients, survivors stayed for an average of 7.67 days, and those who died had a mean stay of 5.60 days (*p* = 0.010). In the group with both sepsis and ARF, survivors had a mean stay of 8.55 days, while those who died had a mean stay of 5.88 days (*p* = 0.043). The clinical presentations are summarized in Table 1.

**Table 1.** Clinical presentation and frequency of in-hospital outcomes (inotropes used, requirement of mechanical ventilation, outcome) of patients diagnosed with sepsis, ARF and both sepsis and ARF.

	Sepsis	ARF	Both sepsis and ARF
No of cases	115	99	43
Male	59	44	19
Female	56	55	24
Mean age	63.43 ± 18.67	65.42 ± 18.65	61.23 ± 19.61
Outcome: -Survive:	75	67	20
Death:	27	25	17
LAMA:	13	7	6
Inotropes used			
Yes:	70	52	31
No:	45	46	12
Mechanical ventilation			
Required:	54	43	31
Not required:	61	56	12
Mean days of mechanical ventilation	1.83	1.58	2.84
Mean days of hospital stay			
For survive:	8.48	7.67	8.55
FOR death:	6.37 (*p* = 0.015)	5.60 (*p* = 0.010)	5.88 (*p* = 0.043)

### For patients diagnosed with sepsis

**Figure 1.**
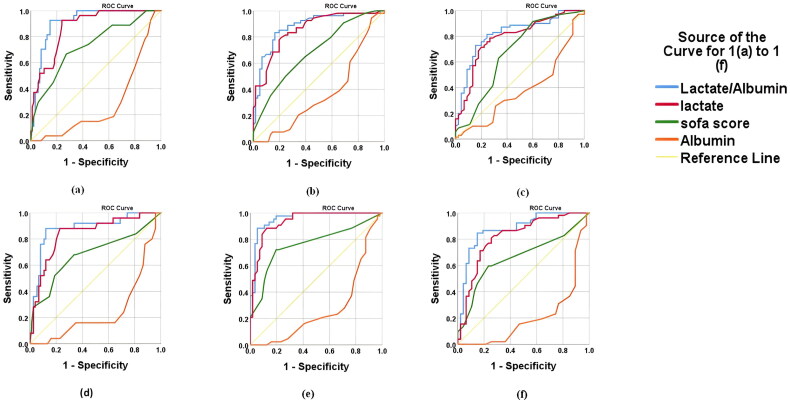
ROC – AUC curve for lactate albumin ratio, lactate alone, albumin alone and SOFA score for (a) mortality in sepsis; (b) need for mechanical ventilation in sepsis; (c) need for inotropes in sepsis; (d) mortality in ARF; (e) need for mechanical ventilation in ARF; (f) need for inotropes in ARF. (note: ROC-AUC of albumin is negatively associated with each of the hospital outcome). The values of AUC of each variable are mentioned in detail in Table 2 for ROC-AUC curve of (a), (b) and (c) for cases of sepsis where as in Table 3 for ROC-AUC curve of 1(e), 1(f) and 1(g) for cases of ARF.

The ROC-AUC curve analysis for Lactate-Albumin Ratio(LAR), Lactate alone, albumin alone and SOFA score for predicting mortality, mechanical ventilation status and use of inotropes is shown in Figure 1 and Table 2.

**Table 2.** AUC value with 95% CI, sensitivity and specificity of lactate/albumin ratio, lactate alone and SOFA score for predicting in hospital outcomes such as mortality, need of inotropes and requirement of mechanical ventilation among sepsis cases.

	AUROC (with 95% CI)	Cut off value	Sensitivity	Specificity	Youden index	*p* Value (Delong test) comparing AUC of LAR with other markers
For mortality						
LAR	0.914 ± 0.038	1.78	92.6%	85.2%	0.778	
Lactate alone	0.873 ± 0.046	4.65	92.6%	76.1%	0.687	0.24 (>0.05)
Albumin alone	0.715 ± 0.061	2.45	55.6%	79.5%	0.351	0.003 (<0.05)
SOFA score	0.730 ± 0.065	>7	66.7%	72.7%	0.394	0.005 (<0.05)
For need of mechanical ventilation						
LAR	0.881 ± 0.045	1.28	83.3%	83.6%	0.669	
Lactate alone	0.857 ± 0.049	4.05	79.6%	78.7%	0.583	0.31 (>0.05)
Albumin alone	0.631 ± 0.056	2.65	46.3%	72.1%	0.184	0.000 (<0.05)
SOFA score	0.689 ± 0.068	>6	64.8%	39.3%	0.225	0.001 (<0.05)
For requirement of inotropes						
LAR	0.819 ± 0.055	1.07	80%	75.6%	0.557	
Lactate alone	0.789 ± 0.059	2.95	78.6%	73.3%	0.499	0.29 (>0.05)
Albumin alone	0.614	2.85	44.3%	75.5%	0.198	0.001 (<0.05)
SOFA score	0.672 ± 0.069	>4	91.6%	40%	0.314	0.009 (<0.05)

*p*-Value demonstrated is for LAR in comparison to other predictive markers such as lactate alone, albumin alone and SOFA score.

The diagnostic accuracy of the lactate/albumin ratio, lactate levels, and the SOFA score for predicting mortality, need of mechanical ventilation and need of inotropes in patient’s sepsis, acute respiratory failure and both was assessed using the area under the receiver operating characteristic (AUROC) curve, sensitivity, specificity, and Youden index.

### For sepsis

The AUC for the lactate/albumin ratio was 0.914 (95% confidence interval (CI): 0.876–0.952), with a cutoff value of 1.78 for predicting mortality. ROC-AUC curve demonstrated a high sensitivity of 92.6% and specificity of 85.2%, yielding a Youden index of 0.778, indicating excellent prognostic performance of LAR for predicting mortality in comparison to lactate alone, albumin alone and SOFA score as shown in Figure 1a and Table 2. The lactate/albumin ratio showed an AUC of 0.881 (95% CI: 0.836–0.926) with a cutoff value of 1.28 for need of intubation. It had a sensitivity of 83.3% and a specificity of 83.6%, yielding a Youden index of 0.669, suggesting strong predictive value for the requirement of requirement of mechanical ventilation in comparison to lactate alone, albumin alone and SOFA score as shown in Figure 1b and Table 2. The lactate/albumin ratio showed an AUC of 0.819 (95% CI: 0.764–0.874) with a cutoff value of 1.07 for need of inotropes. It demonstrated a sensitivity of 80% and a specificity of 75.6%, resulting in a Youden index of 0.557. This indicates a relatively strong predictive value for the need for inotropes in comparison to lactate alone, albumin alone and SOFA score as shown in Figure 1c and Table 2.

### For ARF

The lactate/albumin ratio demonstrated an AUC of 0.878 (95% CI: 0.829–0.927) with a cutoff value of 1.978 for mortality. It had a sensitivity of 88% and a specificity of 87.8%, resulting in a Youden index of 0.88, indicating LAR as better prognostic marker for predicting mortality in comparison to lactate alone (AUC =0.838, 95% CI: 0.782–0.894), SOFA score (AUC = 0.682, 95% CI: 0.608–0.756) among patient diagnosed with ARF as shown in Figure 1d and Table 3.

**Table 3.** AUC value with 95% CI, sensitivity and specificity of lactate/albumin ratio, lactate alone and SOFA score for predicting in hospital outcomes such as mortality, need of inotropes and requirement of mechanical ventilation among ARF cases.

	AUROC with 95% CI	Cut off value	Sensitivity	Specificity	Youden index	*p* Value (Delong test) comparing AUC of LAR with other markers
For mortality						
LAR	0.878 ± 0.049	1.978	88%	87.8%	0.88	
Lactate alone	0.838 ± 0.056	4.65	88%	77%	0.65	0.28 (>0.05)
Albumin alone	0.746 ± 0.061	2.55	60%	86.5%	0.465	0.045 (<0.05)
SOFA score	0.682 ± 0.074	>7	68%	66.2%	0.34	0.007 (<0.05)
For need of mechanical ventilation						
LAR	0.958 ± 0.029	1.104	97.7%	80.4%	0.78	
Lactate alone	0.936 ± 0.036	4.05	88.4%	81.5%	0.7	0.26 (>0.05)
Albumin alone	0.724 ± 0.052	2.65	65.1%	83.4%	0.485	0.000 (<0.05)
SOFA score	0.763 ± 0.066	>6	72.1%	80.4%	0.525	0.001 (<0.05)
For requirement of inotropes						
LAR	0.876 ± 0.050	1.18	84.6%	85.1%	0.7	
Lactate alone	0.816 ± 0.060	3.15	80.8%	74.5%	0.55	0.14 (>0.05)
Albumin alone	0.782 ± 0.046	2.75	54.3%	89.2%	0.435	0.049 (<0.05)
SOFA score	0.661 ± 0.075	>4	59.6%	76.6%	0.36	0.000 (<0.05)

*p*-Value demonstrated is for LAR in comparison to other predictive markers such as lactate alone, albumin alone and SOFA score.

The lactate/albumin ratio demonstrated an AUC of 0.958 (95% CI: 0.929–0.987) with a cutoff value of 1.104 for requirement of mechanical ventilation. It had a high sensitivity of 97.7% and a specificity of 80.4%, resulting in a Youden index of 0.78, indicating LAR as better prognostic marker for the requirement of mechanical ventilation as shown in Figure 1e and Table 3.

The lactate/albumin ratio demonstrated an AUC of 0.876 (95% CI: 0.826–0.926) with a cutoff value of 1.1826. It had a sensitivity of 84.6% and a specificity of 85.1%, resulting in a Youden index of 0.7, indicating LAR as better marker for predicting the need for inotropes among patients diagnosed with ARF as shown in Figure 1f and Table 3.

### For sepsis and ARF

**Figure 2.**
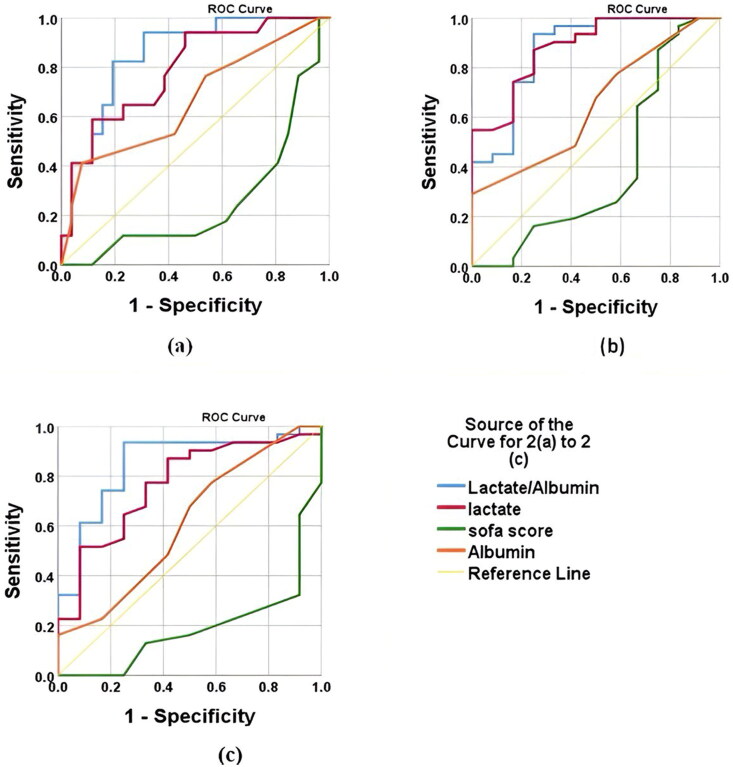
ROC-AUC curve for LAR, lactate alone, SOFA score, albumin alone in patient diagnosed with both sepsis and ARF cases for (a) mortality, (b) need of mechanical ventilation, (c) need of inotropes. (note: ROC-AUC of albumin is negatively associated with each of the hospital outcome). The values of AUC of each variable are mentioned in detail in Table 4.

The lactate/albumin ratio demonstrated an AUC of 0.853 (95% CI: 0.771–0.935) with a cutoff value of 2.14. It showed a sensitivity of 82.4% and a specificity of 80.8%, yielding a Youden index of 0.63, indicating LAR as a better prognostic marker for predicting mortality among cases diagnosed with both sepsis and ARFas shown in Table 4 and Figure 2a.

**Table 4.** AUC value with 95% CI, sensitivity and specificity of lactate/albumin ratio, lactate alone and SOFA score for predicting in hospital outcomes such as mortality, need of inotropes and requirement of mechanical ventilation among patient diagnosed with both sepsis and ARF.

	AUROC with 95% CI	Cut off value	Sensitivity	Specificity	Youden index	*p* Value (Delong test) comparing AUC of LAR with other markers
For mortality						
LAR	0.853 ± 0.082	2.14	82.4%	80.8%	0.63	
Lactate alone	0.790 ± 0.096	5.35	64.7%	77%	0.42	0.26 (>0.05)
Albumin alone	0.734 ± 0.091	2.45	52.9%	84.6%	0.38	0.04 (<0.05)
SOFA score	0.664 ± 0.115	>9	41.2%	92.3%	0.34	0.046 (<0.05)
For need of mechanical ventilation						
LAR	0.874 ± 0.076	1.20	93.5%	75%	0.69	
Lactate alone	0.883 ± 0.073	4.15	87.1%	75%	0.62	0.45 (>0.05)
Albumin alone	0.602 ± 0.093	2.65	51.9%	66.7%	0.17	0.005 (<0.05)
SOFA score	0.649 ± 0.116	>9	77.4%	41.7%	0.19	0.015 (<0.05)
For requirement of Inotropes						
LAR	0.849 ± 0.083	1.20	93.5%	75%	0.69	
Lactate alone	0.766 ± 0.101	3.35	90.3%	50%	0.41	0.19 (>0.05)
Albumin alone	0.710 ± 0.0825	2.75	54.8%	65.2%	0.2914	0.084 (>0.05)
SOFA score	0.609 ± 0.119	>7	67.7%	50%	0.18	0.014 (<0.05)

*p*-Value demonstrated is for LAR in comparison to other predictive markers such as lactate alone, albumin alone and SOFA score.

The lactate/albumin ratio had an AUC of 0.874 (95% CI: 0.798–0.950) with a cutoff value of 1.20. It demonstrated a sensitivity of 93.5% and a specificity of 75%, yielding a Youden index of 0.69 for mechanical ventilation. This indicates a strong ability to predict the need for mechanical ventilation. The lactate level showed an AUC of 0.883 (95% CI: 0.810–0.956) with a cutoff value of 4.15. It had a sensitivity of 87.1% and a specificity of 75%, resulting in a Youden index of 0.62, suggesting it is a reliable predictor of mechanical ventilation, though slightly less effective than the lactate/albumin ratio. The SOFA score demonstrated an AUC of 0.649 (95% CI: 0.533–0.765) with a cutoff value of >9. It had a sensitivity of 77.4% and a specificity of 41.7%, with a Youden index of 0.19 as shown in Table 4 and Figure 2b. This indicates that the SOFA score is less effective in predicting the need for mechanical ventilation compared to the lactate/albumin ratio and lactate levels.

The lactate/albumin ratio showed an AUC of 0.849 (95% CI: 0.766–0.932) with a cutoff value of 1.20. It had a sensitivity of 93.5% and a specificity of 75%, resulting in a Youden index of 0.69. This indicates strong predictive value for the requirement of inotropes. The lactate level demonstrated an AUC of 0.766 with a cutoff value of 3.35. It had a sensitivity of 90.3% but a lower specificity of 50%, yielding a Youden index of 0.41. This suggests it is a reliable predictor but less specific than the lactate/albumin ratio. The SOFA score showed an AUC of 0.609 (95% CI: 0.665–0.867) with a cutoff value of >7. It had a sensitivity of 67.7% and a specificity of 50%, with a Youden index of 0.18, indicating that the SOFA score is a less effective predictor of the need for inotropes compared to the lactate/albumin ratio and lactate levels as shown in Table 4 and Figure 2c.

## Discussion

### Sepsis cases

**Figure 3.**
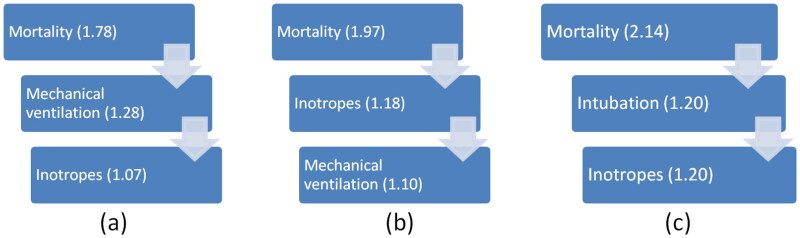
Progression of disease severity as per increasing cut-off value of LAR in (a) sepsis, (b) ARF, (c) both sepsis and ARF. The arrows in this figure demonstrate decreasing trend of cut off value of LAR as the disease severity decreases.

Among sepsis patients, higher SOFA scores, LAR, and Lactate values, along with lower albumin levels, were associated with poor in-hospital outcomes, including a greater need for inotropes and mechanical ventilation. The ROC-AUC curve, demonstrated an increasing cut-off value for LAR in relation to the need for inotropes, mechanical ventilation, and mortality. This progression indicates that, as LAR increases, patients first require inotropes, then mechanical ventilation, and ultimately face higher mortality as depicted in Figure 3. The finding that increased LAR is associated with increased mortality, MODS, and risk stratification of disease correlates with that of the studies by Wang et al., Lichtenauer et al., Yoon et al., and Makram et al. [2,4–6].

### For in hospital outcome/survival

The AUC of LAR was more and found to be better in predicting mortality in sepsis in comparison to lactate alone (AUC: 0.914 v/s 0.873), SOFA score (AUC: 0.914 v/s 0.730), albumin alone (AUC: 0.914 v/s 0.715). As per Delong test, while comparing AUC,LAR was statistically significant over SOFA score (*p* value < 0.05) and albumin alone (*p* value < 0.05) to predict mortality in sepsis. However, the superiority of AUC of LAR over lactate alone was not statistically significant to predict mortality in sepsis (*p* value > 0.05). These results align with those of studies conducted by Jikyoung et al., Chebl et al., Shadvar et al., and Kabra et al. However, in these studies, LAR was superior to lactate alone as well. However, in these studies, LAR was found statistically superior to lactate alone [7,9,10,27]. However, this finding does not correlate with the study conducted by Iskander et al. which states that albumin is better predictor of motality than LAR and lactate alone [28]. The finding is similar to that of another study by Gharipour et al. which reported that the predictive factor of LAR was equivalent to lactate alone for mortality [3].

### For mechanical ventilation

The AUC of LAR was more and found to be better in predicting need of mechanical ventilation in sepsis in comparison to lactate alone (AUC: 0.881 v/s 0.857), SOFA score (AUC: 0.881 v/s 0.689), albumin alone (AUC: 0.881 v/s 0.631). As per Delong test, while comparing AUC, LAR was statistically significant over SOFA score (*p* value < 0.05) and albumin alone (*p* value < 0.05) to predict need of mechanical ventilation among sepsis cases. However, the superiority of AUC of LAR over lactate alone was not statistically significant (*p* value > 0.05). These findings are consistent with those of Shadvar et al. and Makram et al. However, in these studies LAR was statistically significant to lactate [6,10].

### For inotropes

The AUC of LAR was more and found to be better in predicting requirement of inotropes in sepsis in comparison to lactate alone (AUC: 0.819 v/s 0.789), SOFA score (AUC: 0.819 v/s 0.672), albumin alone (AUC: 0.819 v/s 0.614). As per Delong test, while comparing AUC, LAR was statistically significant over SOFA score (*p* value < 0.05) and albumin alone (*p* value < 0.05) to predict need of inotropes. However, the superiority of LAR over lactate was not statistically significant (*p* value > 0.05). Aygun et al. also found that LAR was superior to lactate alone for predicting the need for inotropes but the statistical significance in this study was not established as AUC was not compared in this study [29].

The statistical test in our study didn’t suggest that LAR is superior to lactate alone in predicting in hospital outcomes (mortality, need of mechanical ventilation and inotropes) among sepsis cases. Thus, lactate alone can serve as an effective prognostic marker, similar to LAR in cases of sepsis. However other studies have shown that LAR is statistically superior prognostic marker compared to lactate alone [7,9,10,27]. This conflicting results warrants further investigation through studies with larger sample sizes and a meta-analysis is needed to strengthen the evidence and evaluate whether the combination of lactate and albumin truly provides additional value.

### For hospital stay

Among surviving patients, the SOFA score (*r*^2^ = 0.292) was the best predictor of hospital stay duration, whereas the lactate albumin ratio, lactate alone, and albumin alone had *R*^2^ < 1. For patients who did not die among SOFA score, Lactate Albumin Ratio, lactate alone, and albumin alone had a significant correlation (*r*^2^ < 0.1). These findings contradict those of Gharipour et al., Chebl et al., Shadvar et al., Kabra et al., and Iskandar et al. who indicated that LAR is a predictor of hospital stay duration [3,9,10,27,30].

However, LAR was a better predictor than lactate or albumin alone. Lactate alone also had prognostic significance, as higher lactate levels were associated with in-hospital mortality, need for inotropes, and need for mechanical ventilation, which aligns with the findings of Chambers et al., Chen et al., Krishna et al., Mikkelsen et al., and Zhiqiang et al. [28,31–34].

Similarly, low albumin levels were associated with increased mortality, need for inotropes, and mechanical ventilation, which correlates with Cao et al., Caironi et al., Delaney et al., Kendall et al., Takegawa et al., and Yin et al. where low albumin was associated with poor in-hospital outcomes and albumin resuscitation was associated with better in-hospital outcome [35–40].

### Acute Respiratory failure cases

Similar to sepsis, in ARF patients, a higher SOFA score, LAR, Lactate values, and lower albumin levels were linked to poor in-hospital outcomes requiring inotropes and mechanical ventilation. The ROC-AUC curve indicated an increasing cut-off value for mechanical ventilation, requirement of inotropes, and mortality as 1.98, 1.18 and 1.10 respectively. Unlike sepsis, in ARF with disease progression, as the value of LAR increases at first, mechanical ventilation is needed, then with further progression of disease, inotropes are required, followed by a further increase in the value of LAR, indicating that disease progression indicates mortality. This finding correlates with that of Lu et al. where LAR was found to be an important predictive marker of mortality and poor in-hospital outcomes [24].

### For in hospital outcome/survival

The AUC of LAR was more and found to be better in predicting mortality in ARF in comparison to lactate alone (AUC: 0.878 v/s 0.838), SOFA score (AUC: 0.878 v/s 0.682), albumin alone (AUC: 0.878 v/s 0.746). As per Delong test, while comparing AUC, the superiority of LAR over SOFA score (*p* value < 0.05) and albumin alone (*p* value < 0.05) was statistically significant. However, the superiority of AUC of LAR over lactate was not statistically significant (*p* value > 0.05).

### For mechanical ventilation

The AUC of LAR was more and found to be better in predicting requirement of mechanical ventilation in ARF in comparison to lactate alone (AUC: 0.958 v/s 0.936), SOFA score (AUC: 0.958 v/s 0.763), albumin alone (AUC: 0.958 v/s 0.724). As per Delong test, while comparing AUC the superiority of LAR over SOFA score (*p* value < 0.05) and albumin alone (*p* value < 0.05) was statistically significant. However, the superiority of AUC of LAR over lactate was not statistically significant (*p* value > 0.05).

### For inotropes

The AUC of LAR was more and found to be better in predicting need of inotropes in ARF in comparison to lactate alone (AUC: 0.876v/s 0.816), SOFA score (AUC: 0.876 v/s 0.661), albumin alone (AUC: 0.876 v/s 0.782). As per Delong test, while comparing AUC the superiority of LAR over SOFA score (*p* value < 0.05) and albumin alone (*p* value < 0.05) was statistically significant. However, the superiority of AUC of LAR over lactate was not statistically significant (*p* value > 0.05).

Despite apparent trend favoring LAR over lactate alone, the statistical test did not provide enough evidence to confirm that LAR is superior to lactate alone predicting in hospital outcomes (mortality, need of mechanical ventilation and inotropes) in ARF cases. Thus, lactate alone can be used as an effective prognostic marker similar to LAR in ARF cases. However, lack of sufficient studies in ARF cases and the greater AUC of LAR compared to lactate alone although not statistically significant, warrants further evaluation with larger sample sizes.

### For hospital stay

Unlike Sepsis, among patients diagnosed with ARF, regardless of hospital outcome (for both alive and dead patients), among SOFA score, Lactate Albumin Ratio, lactate alone, and albumin alone had a significant correlation (*r*^2^ < 0.1).

However, the role of LAR as a predictor of in-hospital outcomes, use of inotropes, and requirement of mechanical ventilation in ARF have not been explored in previous studies. The role of lactate and albumin and their impact on in-hospital outcomes in ARDS, Pneumonia, and ARF due to COVID-19 have been explored in different studies.

Increased lactate levels were associated with increased mortality and poor in-hospital outcomes, similar to the findings of Kamo et al., Nanda et al., Grezia et al., Hussein et al., and Marazzi et al. where increased lactate levels were associated with increased mortality, in-hospital complications, and longer hospital and ICU stays in ARDS and ARF related to COVID-19 [12–14,41,42].

A decrease in albumin level was associated with increased mortality and poor in-hospital outcomes, similar to the findings of Viasus et al., Su et al., Kheir et al., Hoebaer et al., and Thongprayoon et al. where a decrease in albumin level was associated with increased mortality, in-hospital complications, and longer hospital and ICU stays, and improvement in overall in-hospital outcomes with albumin replacement therapy in ARDS and ARF related to COVID-19 and pneumonia [14,15,43–45].

However, the role of lactate and albumin alone in predicting in-hospital outcomes was good. No study has compared LAR with lactate alone, albumin alone, and SOFA score. In our study, the role of LAR as a predictor for in-hospital outcomes (mortality, need for inotropes, and mechanical ventilation) was better than that of lactate or albumin alone.

### For both sepsis and ARF cases

Among the patients, a higher SOFA score, LAR, Lactate value, and lower albumin level were associated with poor in-hospital outcomes, with more need of inotropes and mechanical ventilation. As per ROC-AUC curve, the cut-off value for LAR was in increasing order for requirement of inotropes, Need of mechanical ventilation, and mortality i.e. 1.20, 1.21, 2.14 were the cut-off values for inotropes requirement, mechanical ventilation status, and as mortality indicator respectively. Thus, this increasing order of cut-off values of LAR for different in-hospital outcomes and step-wise rise in LAR with progression of the disease requiring inotropes as disease progresses first, followed by mechanical ventilation, and finally mortality.

### For in hospital outcome/survival

The LAR was more and found to bebetter in predicting mortality among patients diagnosed with both sepsis and ARF in comparison to lactate alone (AUC: 0.853v/s 0.790), SOFA score (AUC: 0.853 v/s 0.664), albumin alone (AUC: 0.853 v/s 0.734). As per Delong test, while comparing AUC, the superiority of AUC of LAR over SOFA score (*p* value < 0.05) and albumin alone (*p* value < 0.05) was statistically significant. However, the superiority of AUC of LAR over lactate was not statistically significant (*p* value > 0.05).

### For mechanical ventilation

The LAR was more and found to be better in predicting need of mechanical ventilation among patients diagnosed with both sepsis and ARF in comparison toSOFA score (AUC: 0.874 v/s 0.649), albumin alone (AUC: 0.874 v/s 0.602). As per Delong test, while comparing AUC the superiority of AUC of LAR over SOFA score (*p* value < 0.05) and albumin alone (*p* value < 0.05) was statistically significant. Although lactate alone (AUC: 0.874v/s 0.883) has slight superior AUC than LAR in predicting need of mechanical ventilation. However, the superiority of AUC of lactate alone was not statistically significant (*p* value > 0.05).

### For inotropes

The LAR was more and found to be better in predicting need of inotropes among patients diagnosed with both sepsis and ARF in comparison to lactate alone (AUC: 0.849 v/s 0.810), SOFA score (AUC: 0.849 v/s 0.609), albumin alone (AUC: 0.849 v/s 0.710). As per Delong test, while comparing AUC the superiority of AUC of LAR over SOFA score (*p* value < 0.05) and albumin alone (*p* value < 0.05) was statistically significant. However, the superiority of AUC of LAR over lactate was not statistically significant (*p* value > 0.05).

The AUC of LAR (cut-off value 1.20) was 0.849, with a sensitivity, specificity and Youden index of 93.5%, 75%, and 0.69 respectively. The AUC for LAR (0.849) was better than that of albumin alone (0.810), lactate alone (0.766), and SOFA score (0.609), with the Youden index of LAR (0.69) being superior to that of lactate alone (0.41) and SOFA score (0.18), suggesting that LAR has better sensitivity and specificity for the need for inotropes in patients with sepsis and ARF than the SOFA score and lactate alone.

### For hospital stay

Among live patients, SOFA score (*r*^2^ = 0.223) was a better predictor of hospital stay duration than LAR, lactate alone, and albumin alone (*r*^2^ < 0.1). None of these markers had showed a significant correlation (*r*^2^ < 0.1) in deceased patients.

No previous studies have investigated the role of LAR, lactate alone, albumin alone, and SOFA score as predictive marker for in-hospital outcomes among patients diagnosed with both sepsis and ARF, which signifies the importance of LAR as a better predictive marker than lactate alone, albumin alone, and SOFA score in predicting in-hospital outcomes and requires a larger sample size evaluation.

LAR as a prognostic marker was not statistically superior to lactate alone predicting in hospital outcomes (mortality, need of mechanical ventilation and inotropes) in cases diagnosed with sepsis and ARF. Thus, lactate alone can be used as an effective prognostic marker similar to LAR in sepsis and ARF cases. However, the greater AUC of LAR compared to lactate alone although not statistical significant and considering our study being novel study to evaluate LAR as prognostic marker among patients diagnosed with sepsis and ARF, warrants further evaluation with larger sample sizes.

Limitations of the study and future direction:

1. Only the initial arterial samples for ABG, venous samples for serum albumin, and SOFA scores were obtained at presentation, with no serial evaluations.
2. While evaluation of LAR as prognostic marker in patients with both sepsis and ARF is a newer approach, larger sample size and multi-centric is required to further strengthen the evidence.
3. The study didn’t account for renal function which would have affected lactate clearance and consequently, affecting LAR. This requires further evaluation and presents an opportunity for new research.
4. Although patients having impaired liver function or chronic liver disease were excluded from the study, other comorbidities and medication usage may have influenced LAR.
5. The impacts of Interventions that improve LAR such as albumin supplementation and enhancing Lactate clearance, on patients in hospital outcome can be further studied. These interventions may play significant role in developing guidelines for use of LAR as both prognostic marker and new interventions in sepsis, ARF and combined sepsis and ARF.

## Conclusion

In sepsis, ARF, and combined sepsis and ARF cases, initial LAR was a better predictor of in-hospital outcomes such as in-hospital mortality, need for inotropes, and mechanical ventilation than albumin alone, and SOFA scores. Although AUC of LAR was greater than lactate alone in predicting hospital outcome in sepsis, ARF and combined sepsis and ARF cases, it was not statistically significant. While the Lactate-to-Albumin Ratio (LAR) proved to be a better predictor, other markers such as elevated lactate levels, higher SOFA scores, and lower albumin concentrations were also linked to poor in-hospital outcomes. However, the SOFA score was the only predictor of duration of hospital stay among surviving sepsis patients. None of the markers i.e. LAR, lactate alone, SOFA score and Albumin level were effective at predicting the duration of hospital stay among cases of ARF or combined sepsis and ARF regardless of in hospital outcome. An increase in the LAR was directly associated with disease progression, evidenced by a step-ladder incresase in the cut-off values of LAR that correlated with the need for inotropes, mechanical ventilation and higher mortality rates among patients with sepsis, ARF or combination of both. For patients with both sepsis and ARF, a lack of sufficient studies warrants a larger sample size to evaluate the role of LAR as a predictive marker for in-hospital outcomes.

## Data Availability

The data generated and analyzed during the study are available from the corresponding author on reasonable request. There are no ethical, privacy and security concerns preventing the release of these data.

## Abbreviations

SOFA: Sequential Organ Failure Assessment
APACHE: Acute Physiology and Chronic Health Evaluation
ARF: Acute Respiratory Failure
LAR: Lactate Albumin Ratio
ABG: Arterial Blood Gas analysis
